# Supplementary material for: Protein language model pseudolikelihoods capture features of in vivo B cell selection and evolution
Source: Brief Bioinform. 2025 Aug 18;26(4):bbaf418. doi: 10.1093/bib/bbaf418 (PMC12360699; doi:10.1093/bib/bbaf418)
Supplement: PLM_manuscript_supplementals_bbaf418 [file plm_manuscript_supplementals_bbaf418.docx]

**Supplementals**


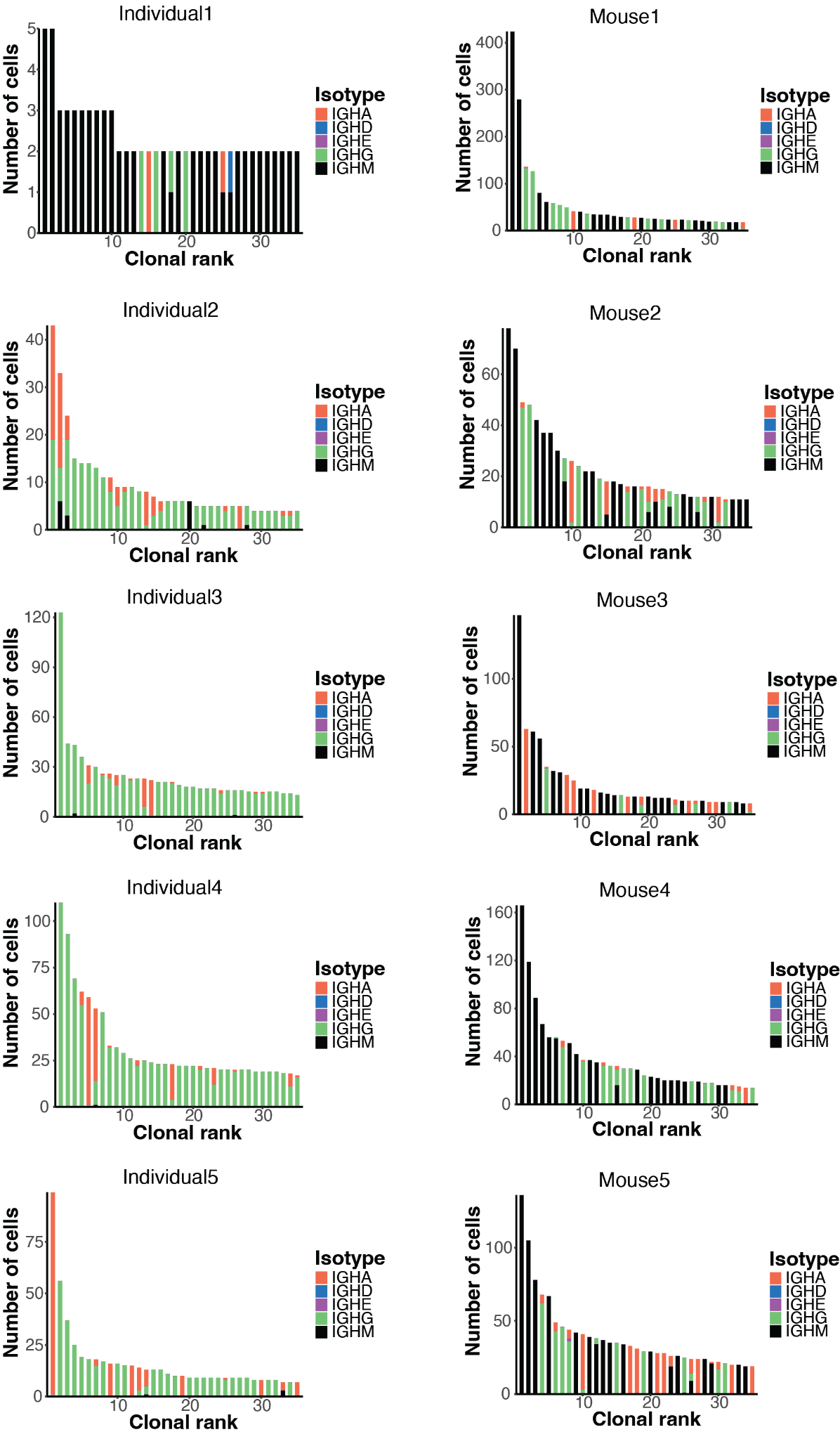


Figure S1. Clonal expansion of all human samples (left) and mouse samples (right) colored on isotype. Ranked bar plots depict the thirty-five most expanded clones of each sample.


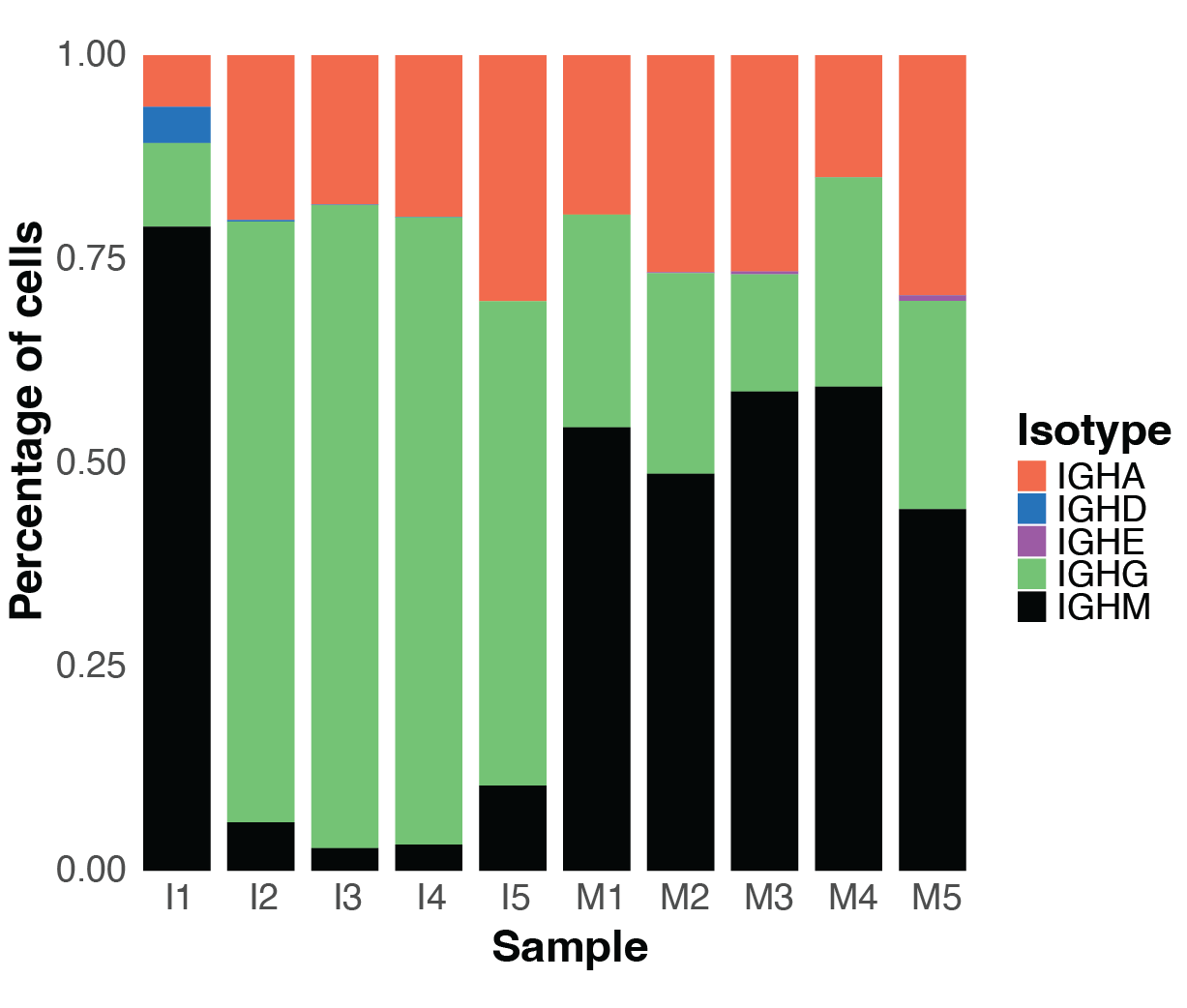


Figure S2. Isotype distribution per sample. I = Individual, M = Mouse.


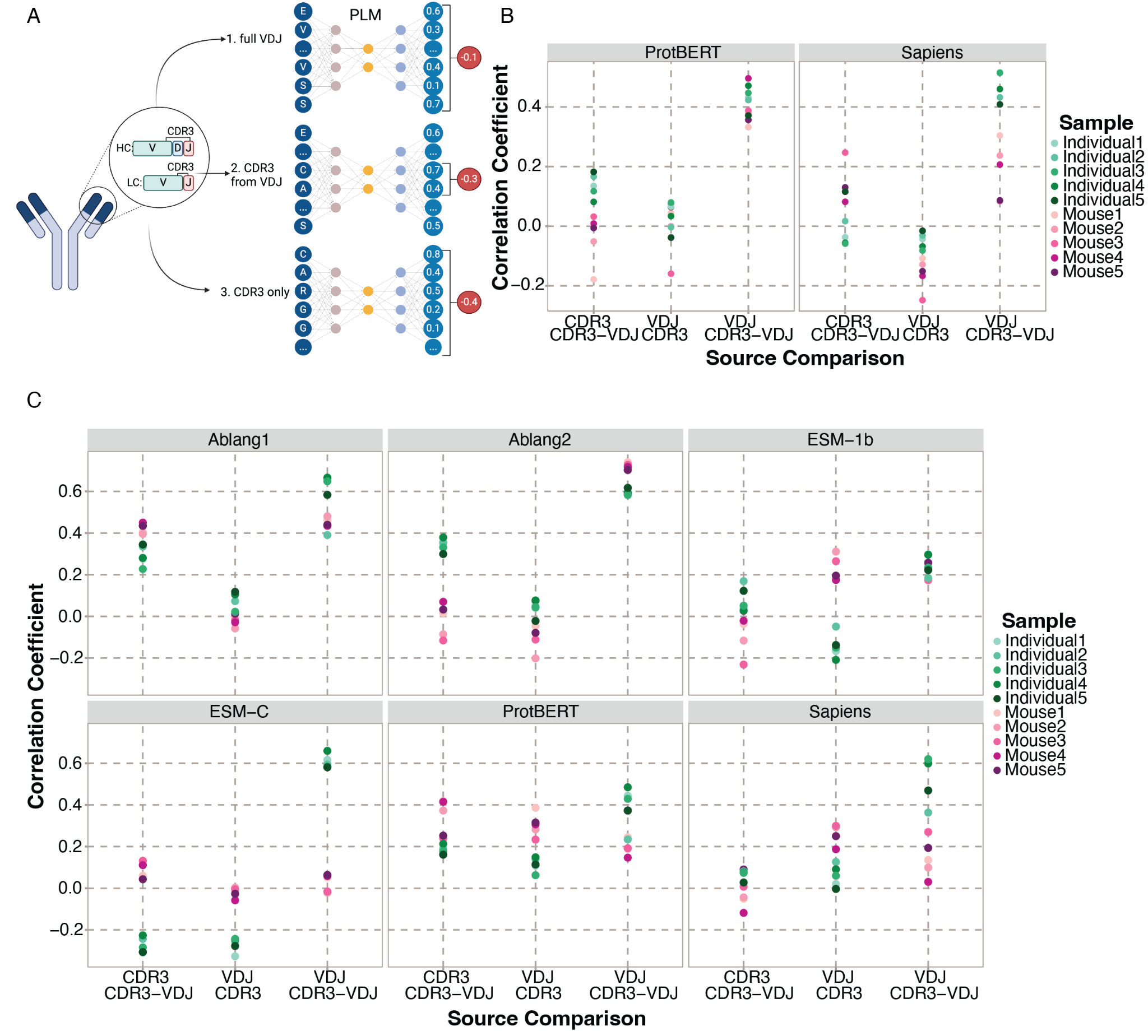


Figure S3. Input source correlations. A) Schematic overview of the three input sources; 1: full VDJ as input and full VDJ used to calculate SP, 2: full VDJ as input but only CDR3 used to calculate SP, 3: CDR3 as input and CDR3 used to calculate SP. Created in BioRender. B) The Pearson Correlation of SPs of the heavy chains between the three sources of input for ProtBERT and Sapiens (other four PLMs are in main Figure 1C). C) The Pearson Correlation of SPs of the light chains between the three sources of input for all PLMs..

**
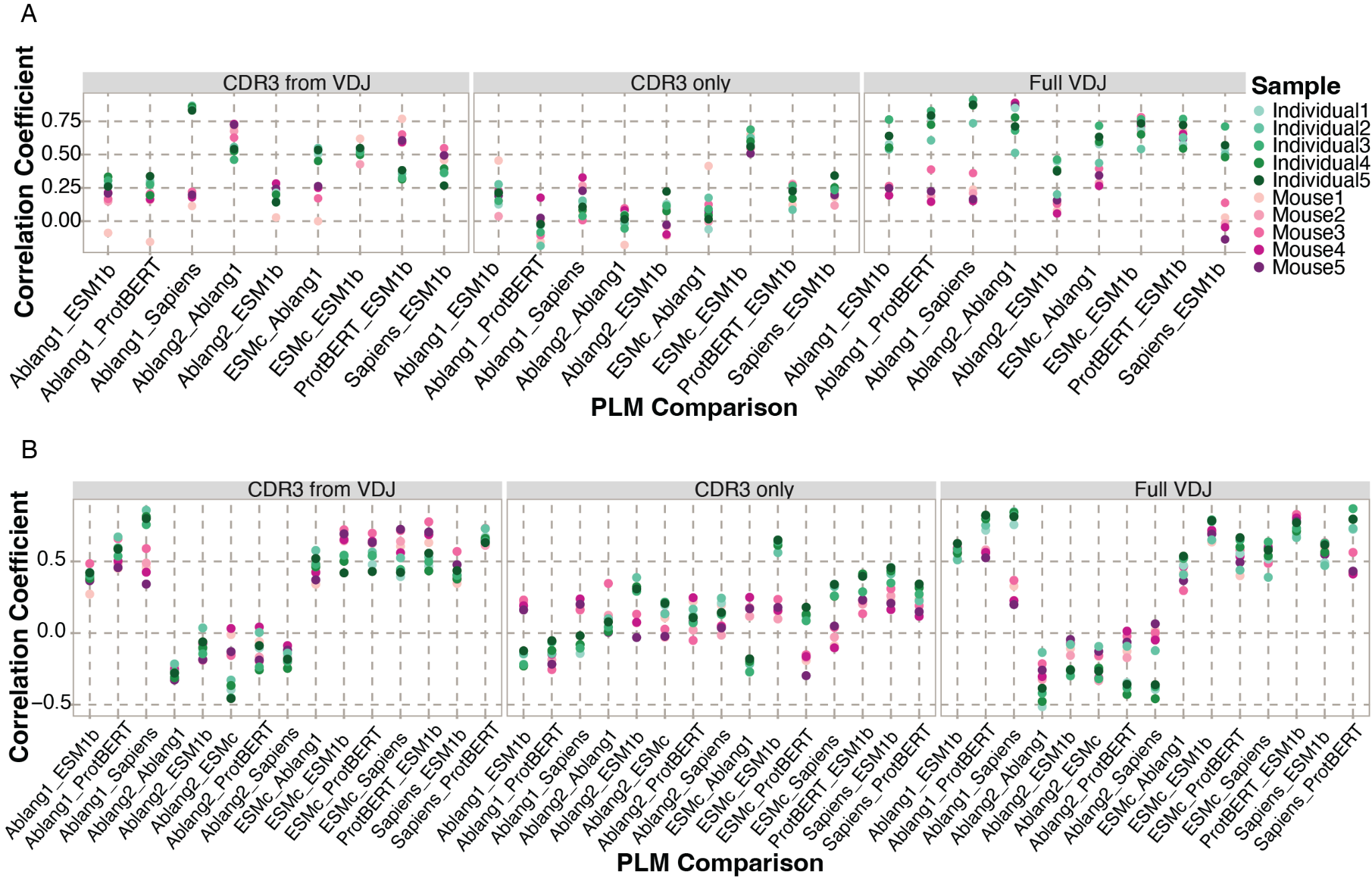
**

Figure S4. Pearson Correlation between SPs of different PLMs. A) Correlation between SPs of the heavy chains calculated with different PLMs for each of the three input sources. B) Correlation between SPs of the light chains calculated with different PLMs for each of the three input sources.


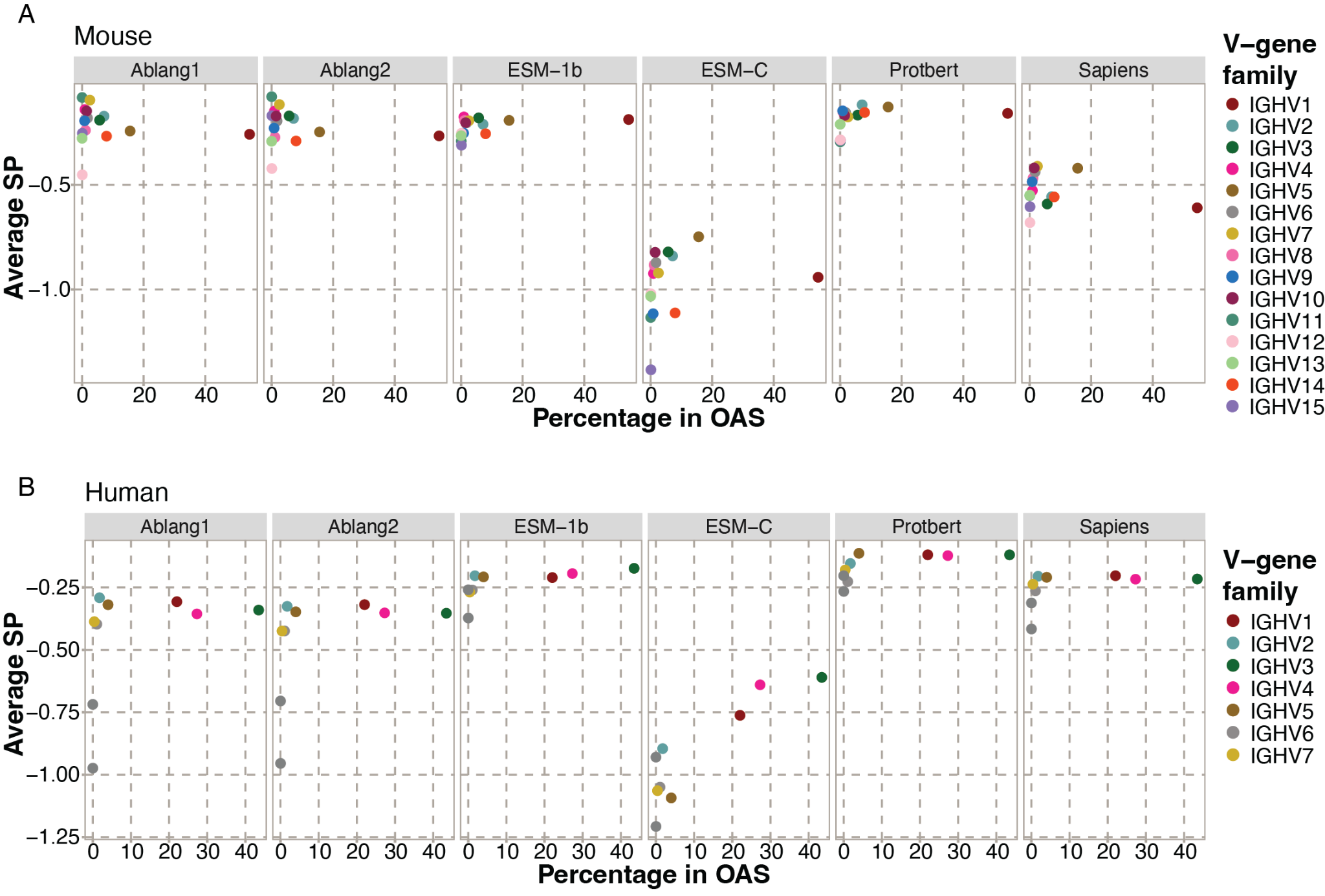


Figure S5. Correlation between SP and isotype frequency in the OAS. A) The average SP per V-gene family and the percentage of unique sequences of this V-gene family in the OAS database for the mouse samples. B) The average SP per V-gene family and the percentage of unique sequences of this V-gene family in the OAS database for the human samples.


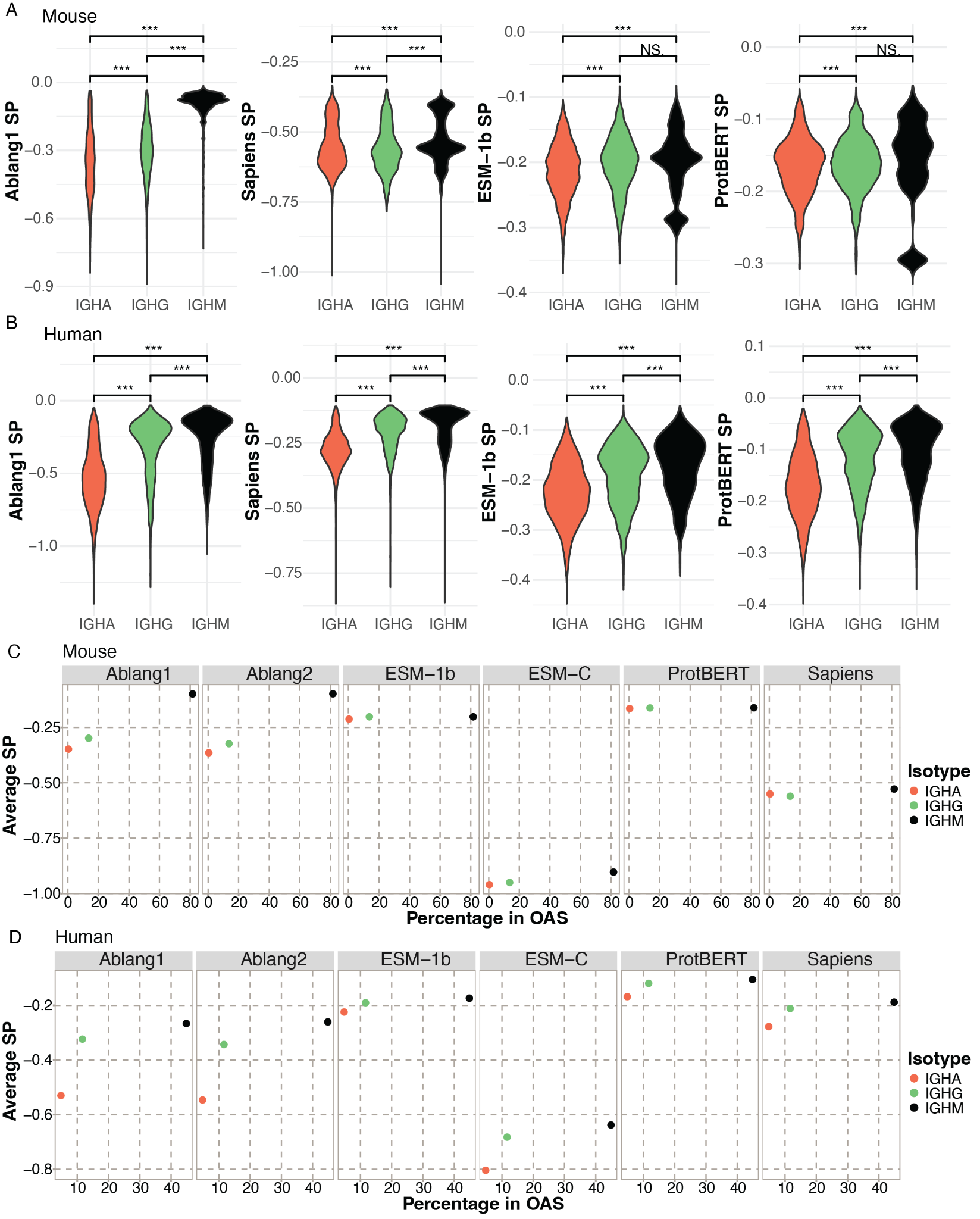


Figure S6. Correlation between SPs and isotypes. A) Distribution of SPs of mouse BCRs from certain isotypes. B) Distribution of SPs of human BCRs from certain isotypes. T-test significance: *** = adjusted p-value below 0.001, NS = non-significant. C) The average SP per isotype and the percentage of unique sequences of this isotype in the OAS database for the mouse samples. D) The average SP per isotype and the percentage of unique sequences of this isotype in the OAS database for the human samples**.**


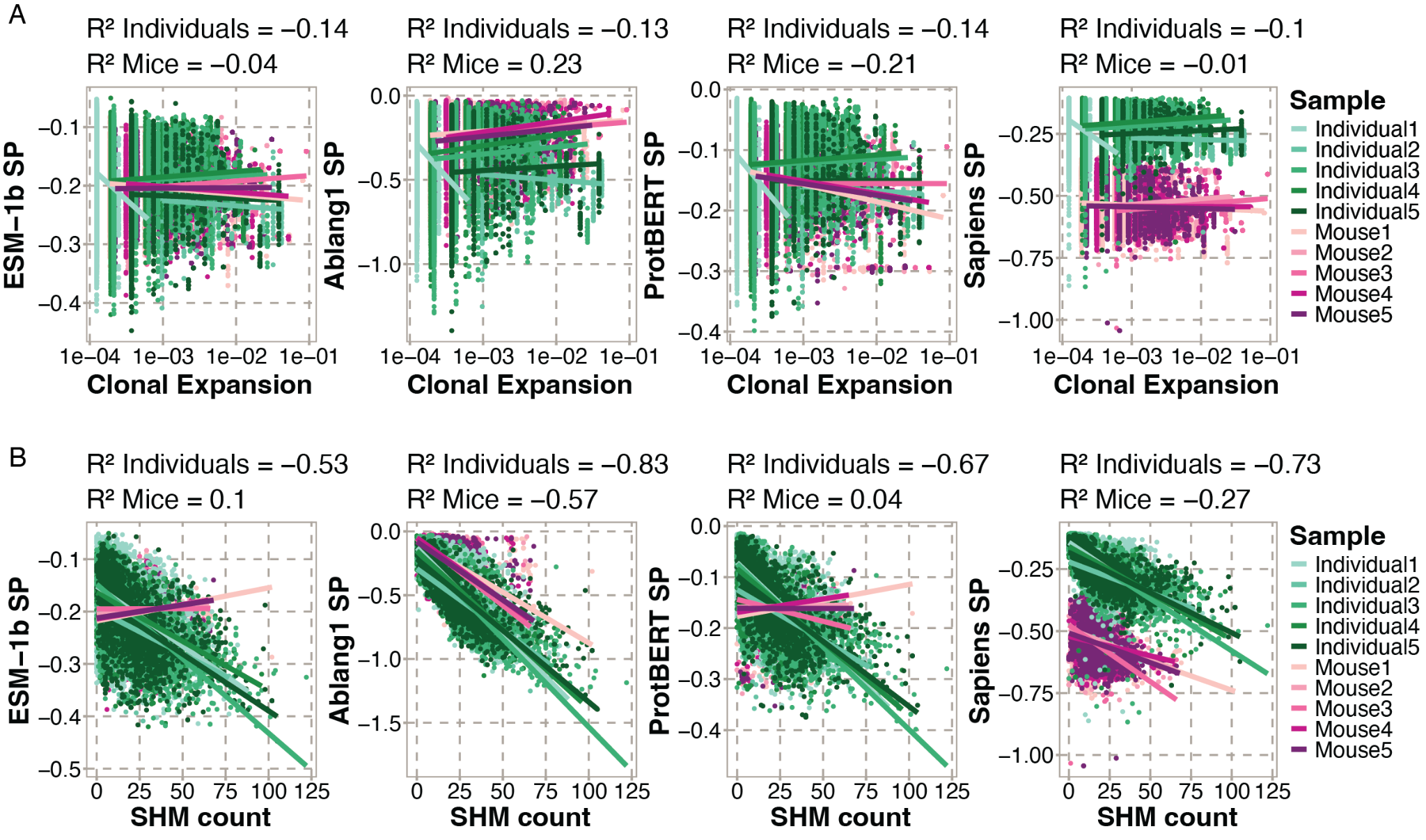


Figure S7. Correlation between SPs and features of B cell repertoire evolution. A) Pearson Correlation between normalized clonal expansion (number of cells per clonotype divided by the sample size) and SP for four PLMs. B) Pearson Correlation between the amount of SHM (hamming distance from the germline) and SP for four PLMs.


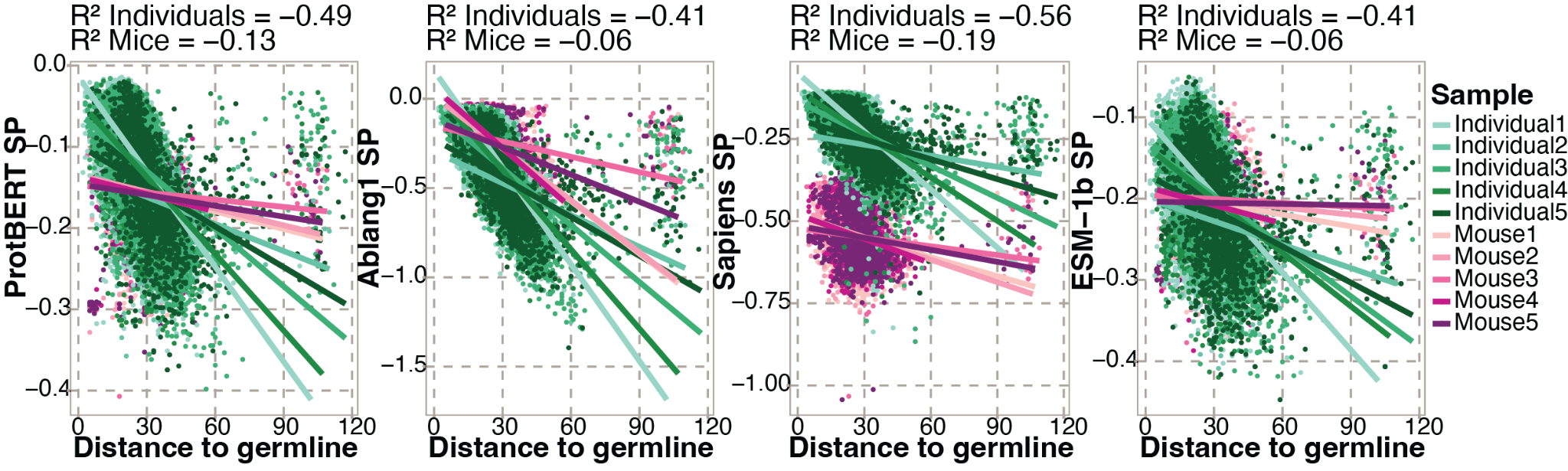


Figure S8. Correlation between SP and the sum of edge lengths to the germline for each heavy chain BCR sequence in the lineage trees of all samples.


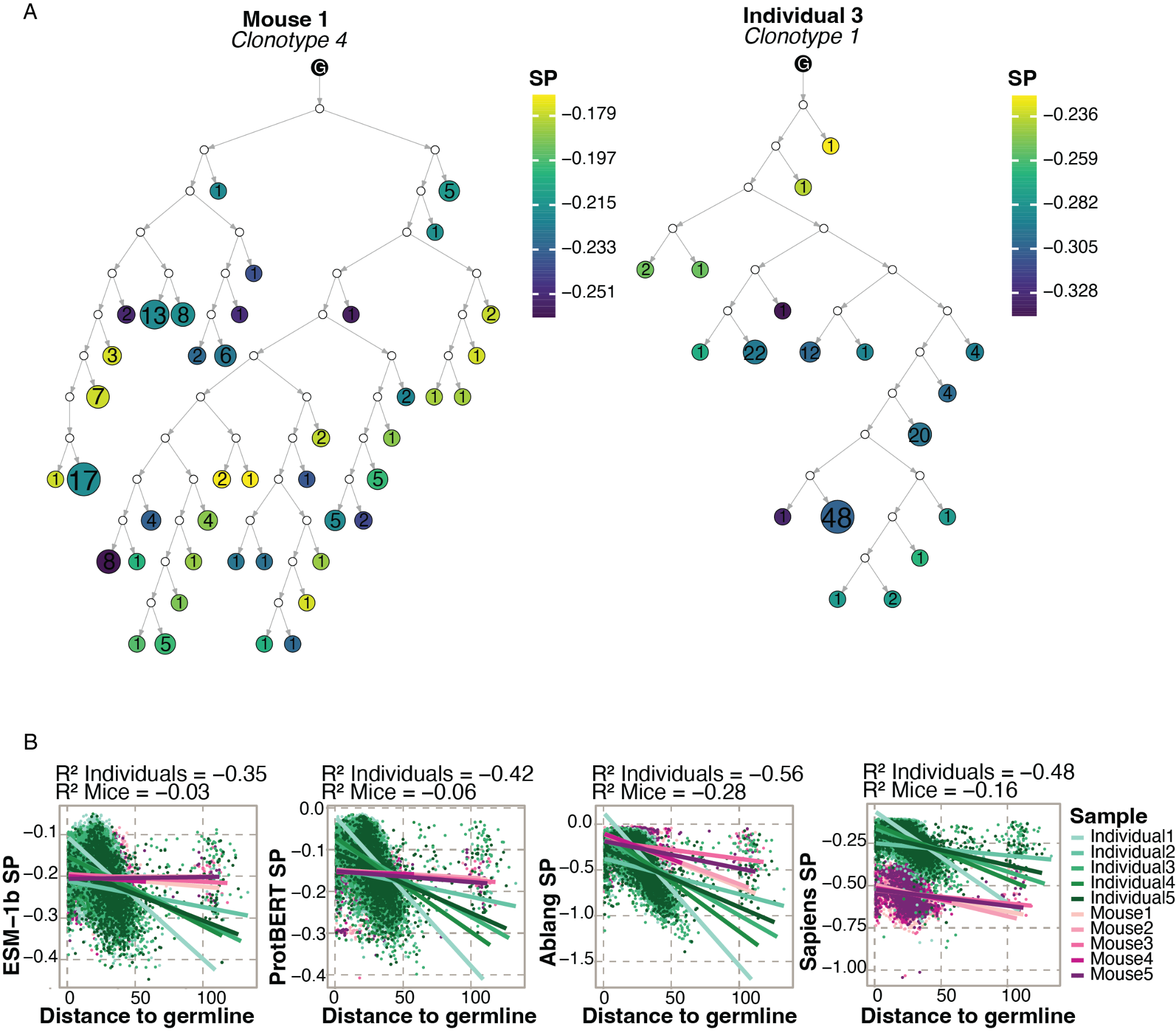


Figure S9. Phylogenetic analysis of the clonal lineages constructed with a maximum parsimony algorithm. A) Representative plots of the lineage trees colored by ESM-1b full-VDJ SP. White nodes represent intermediate nodes recovered by the phylogenetic algorithm. B) Correlation between SP of all PLMs and the total edge length (Levenshtein distance) to the germline for each sequence in all trees.


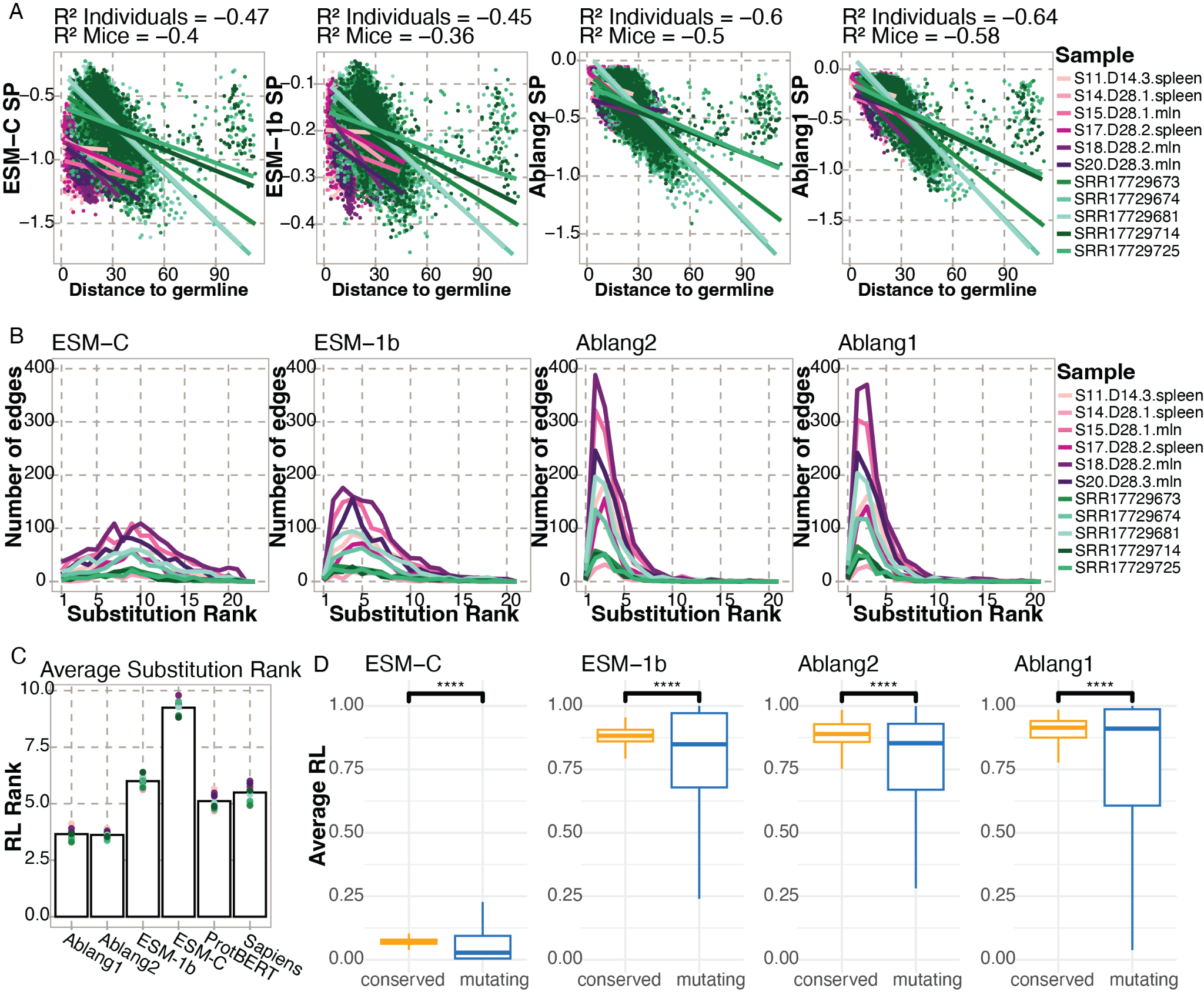


Figure S10. Additional data for lineage tree validation. Purple indicates mouse samples from Mathew et al. Cell Rep, 2022. Green indicates additional human samples from Kim et al. Nature 2022. A) Pearson Correlation between SP and the total edge length to the germline (Levenshtein distance) for each sequence in all trees. B) The RL ranks of the substitutions along the edges of the lineage trees. The average rank is used for edges with multiple substitutions. C) Mean substitution RL rank for each sample (dots) and average of all samples (bars) per PLM. E) Difference in average RL between conserved and mutating residues. T-test significance: **** = adjusted p-value below 0.0001.


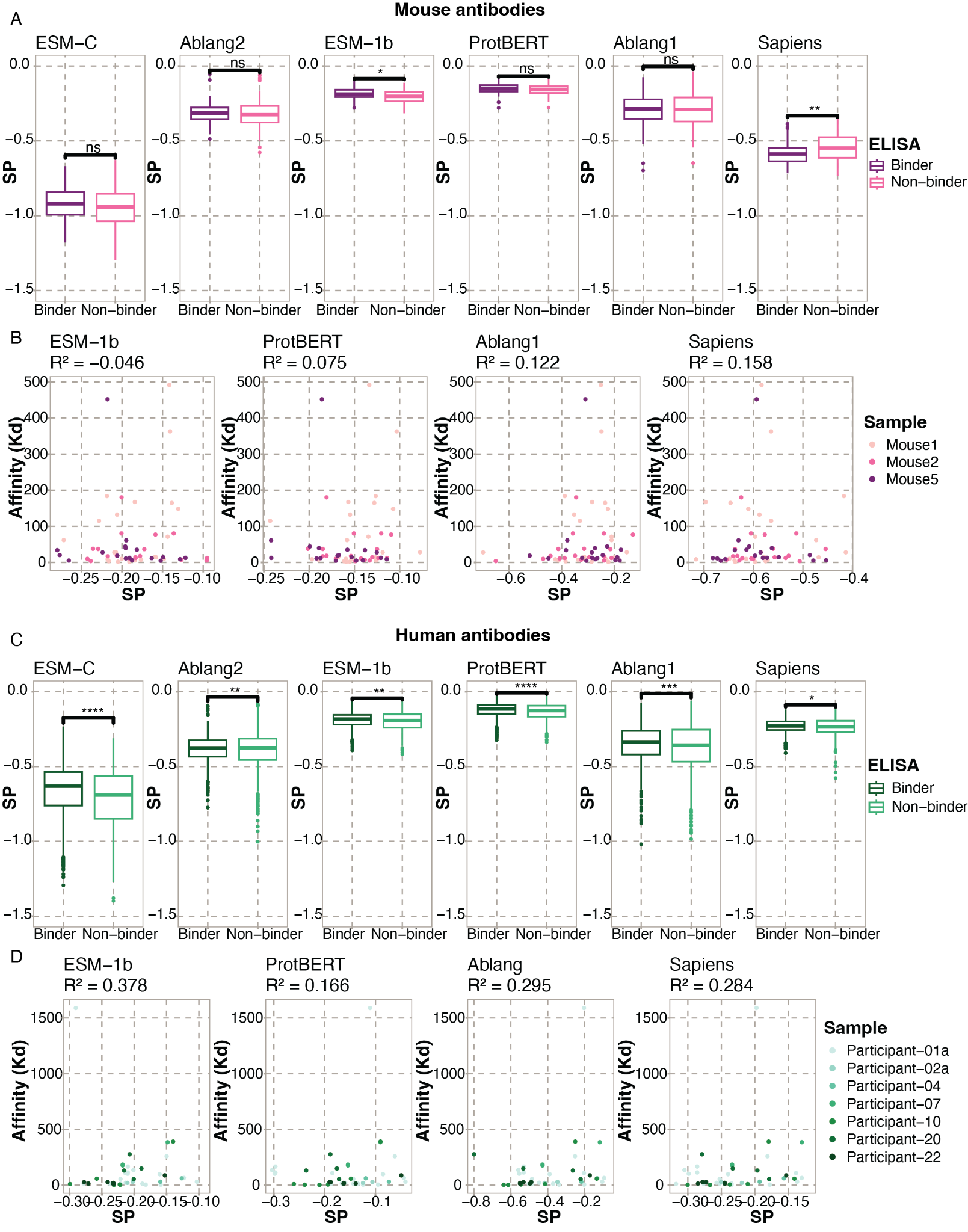


Figure S11. ​​Spearman Correlation of SP with polyclonal antigen-specificity and binding affinity. A) SP and antigen-specificity against Ovalbumin. B) Correlation between binding affinity and SPs for mouse samples. C) SP and antigen-specificity against Sars-COV-2 S protein. D) Correlation between binding affinity and SP for 7 individuals (sample IDs correspond to those of Kim et al.[(26)](https://paperpile.com/c/X6WhRI/M0Gu)). (T-test significance: ns = p > 0.05; * = p ≤ 0.05; ** = p ≤ 0.01; *** = p ≤ 0.001; **** = p ≤ 0.0001)
